# The Prevalence of Bacterial and Fungal Coinfections among Critically Ill COVID-19 Patients in the ICU in Jordan

**DOI:** 10.1155/2022/9992881

**Published:** 2022-10-31

**Authors:** Ayman Daifallah Alsheikh, Mohamed Abdelsalam Abdalla, Mai Abdullah, Hanan Hasan

**Affiliations:** ^1^Department of Medical Laboratory Science, Faculty of Allied Medical Sciences, Zarqa University, Zarqa, Jordan; ^2^Department of Preventive Medicine and Public Health, College of Veterinary Medicine, Sudan University of Science and Technology, Khartoum, Sudan; ^3^Department of Nutrition and Food Technology, Faculty of Agriculture, The University of Jordan, Amman, Jordan; ^4^Department of Pathology, Microbiology and Forensic Medicine, School of Medicine, The University of Jordan, Amman, Jordan; ^5^Mega Labs, Amman, Jordan

## Abstract

**Background:**

Secondary bacterial and fungal coinfections have been reported among critically ill coronavirus disease-19 (COVID-19) patients and are associated with increased disease severity and mortality incidence (MI) rates.

**Aims:**

This study aimed to track bacterial and fungal coinfections among COVID-19 patients in the intensive care unit (ICU) and to assess the impact of these infections on disease prognosis and patient outcomes in Jordan.

**Materials and Methods:**

This was a single-center study that enrolled 46 ICU patients diagnosed with COVID-19. Microbiological and antimicrobial susceptibility results and inflammatory biomarker data were retrospectively analyzed.

**Results:**

The MI rate attributed to bacterial and fungal coinfections was 84.8%, and the highest rate was reported among patients older than 70 years (66.7%). The MI rate related to bacterial coinfections was 95.2%, whereas that of fungal coinfections was 4.8%. The most commonly isolated bacterium in the blood was a coagulase-negative staphylococcus (41%), followed by *Klebsiella pneumoniae* in nasopharyngeal swabs (34%) and *Acinetobacter baumannii* in sputum samples (31%). *Candida* species were the sole cause of fungal coinfections in the studied population. In particular, *Candida albicans* was isolated from 3% of patients with bacteremia, whereas *Candida glabrata* was isolated from 8% of nasopharyngeal swabs. *Klebsiella pneumoniae* was considered the major cause of upper respiratory tract infections (34%). Multifactorial infection was significantly associated with increased MI (*p* value <0.001).

**Conclusion:**

COVID-19 MI is associated with respiratory bacterial/fungal coinfections. The ability to predict bacterial and fungal coinfections in ICU patients may be crucial to their survival and prognosis.

## 1. Introduction

A large proportion of patients with respiratory viral infections develop secondary bacterial and/or fungal coinfections, leading to increased severity and mortality due to the synergistic interaction of microbial pathogenesis and the host immune system [1]. The risk of death among COVID-19 patients increases by more than twofold in the presence of bacterial and fungal coinfections [2]. Additionally, several studies have reported low rates of confirmed bacterial coinfections in COVID-19 patients, which were attributed to the lack of prompt diagnosis and administration of broad-spectrum empirical antibiotics in COVID-19 patients at the time of hospital administration [3].

The exact mechanism by which COVID-19 predisposes patients toward coinfections with other microorganisms is not yet fully understood. However, there are different hypotheses to explain this relationship; one of them is that infected respiratory cells are induced to release anti-inflammatory cytokines [4]; these cytokines inhibit the link between the adaptive and innate immune systems, leading to delayed or inhibited bacterial clearance [4]. Another hypothesis is that the production of viral enzymes such as neuraminidase and sialidase promotes bacterial and fungal colonization [5].

The prevalence of COVID-19 coinfection varies in different situations. The highest prevalence of coinfections has been detected among hospitalized immune-suppressed patients (who were exposed to central lines and mechanical ventilators) and patients who had an underlying history of diabetes or other chronic diseases [6, 7]. Such infection may be caused by a single microbe or multiple microbes [8, 9], with various complications such as severe pneumonia [8], epidermal signs [10], alterations in the gastrointestinal microbiome [11], bacteremia [12], and hospital-acquired bacterial infections in the intensive care unit (ICU) [13]. Additionally, the presence of multidrug-resistant bacteria creates an additional crisis in the treatment of critically ill COVID-19 patients [14]. Complications of coinfections in COVID-19 patients depend on the etiology of coinfected microorganisms, viral load, “severity of viral infection,” and the host immune response to the infection [1].

A computerized tomography (CT) scan plays a vital role among critically ill COVID-19 patients admitted to the ICU because it is used in the monitor of patients, evaluating patient disease severity and prognosis, informing the treating physician about laboratory tests required, and classifying patients into different risk groups [15, 16].

Several studies have reported that clinicians tend to administer empirical broad-spectrum antibiotics to treat or avoid suspected bacterial coinfection [17, 18]. This, in turn, results in many adverse effects that change the normal microflora, leading to the emergence of new antibiotic-resistance mutations [19]. Therefore, it is important to begin empirical therapies to ensure patient survival and then to apply narrower-spectrum antibiotics after receiving microbiological lab results [20, 21]. Therefore, this study aimed to investigate isolated bacterial and fungal coinfections among COVID-19 patients in the ICU and to assess the impact of such coinfections on disease prognosis and patient outcomes in a private hospital in Amman, Jordan.

## 2. Materials and Methods

**Figure 1:**
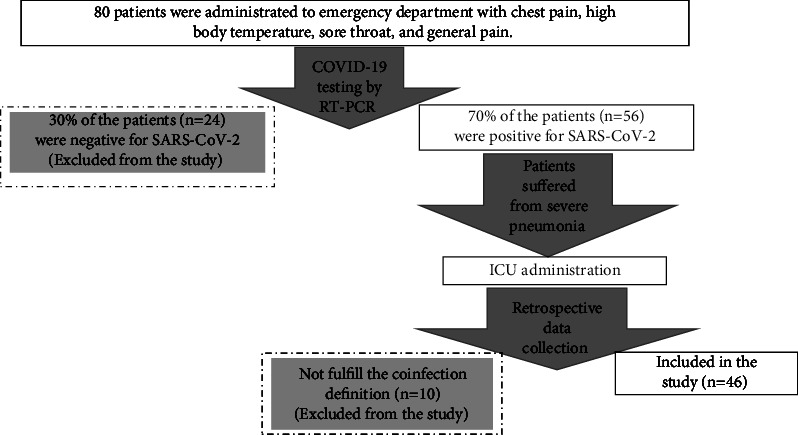
Flow chart for inclusion of the study participants.

Eighty critically ill patients were admitted to the ICU between January 2020 and June 2021 in a private hospital in Amman, Jordan. All of them were assessed for COVID-19 infection by real-time reverse transcription-polymerase chain reaction (RT‒PCR). Only 56 were diagnosed with COVID-19, and they were included in the study. Based on the definition of coinfection, namely, infections that occur ≤48–72 h after hospital admission [22], only 46 patients fulfilled the criterion and were included in the study. In addition, 10 patients were excluded from the study owing to missing data regarding the date of administration and the date of bacterial and fungal growth (Figure 1).

At the time of ICU administration, required clinical, radiological, and microbiological assessments were performed. Bacterial isolates were identified based on colony characteristics and biochemical tests using a VITEK 2 system (bioMérieux)® (USA); fungal isolates were determined by colony morphology and colony characteristics. A galactomannan test to detect bacteremia caused by aspergillosis infections was not performed. Data collected included age, sex, patient outcome (died or survived), treatment provided, coinfection outcomes (upper respiratory infection, lower respiratory infection, bacteremia, and meningitis), and antimicrobial susceptibility results. Additionally, data on inflammatory biomarkers, including C-reactive protein (C-RP), ferritin, d-dimer, procalcitonin (PCT), globulin, white blood cell count (WBC), neutrophil count, and lymphocyte count, were selected to be included in the study to indicate the impact of bacterial and fungal coinfections due to missing data for other parameters of hematological, biochemical, and serological test profiles.

Ethical approval was obtained from the Ethics Committee for Scientific Research (ECSR) at Zarqa University based on the requirement for the protection of human subjects and the ethical principles related to research studies (no. 2/8/2021). Data were analyzed using IBM SPSS Statistics (IBM Corp. Released 2012. IBM SPSS Statistics for Windows, Version 25.0. Armonk, NY: IBM Corp). The chi-square (*χ*^2^) test was performed for categorical variables and the independent samples *t*-test and Mann‒Whitney *U* test for continuous variables; results are presented as the mean and standard deviation. Statistical significance was defined as a *p* value <0.05.

## 3. Results

A total of 46 ICU COVID-19 patients were included in this study. The types of isolated microorganisms for each and patient demographic data (age, sex, and patient outcomes) are presented in Table 1. The major isolated infectious bacteria were *Klebsiella pneumoniae*, *Staphylococcus aureus*, *Enterococcus faecalis, Serratia marcescens, Pseudomonas aeruginosa, Acinetobacter baumannii,* coagulase-negative staphylococci, *Coryneform bacilli*, and *Staphylococcus epidermidis.* Among the isolated fungi, only two species of *Candida* were detected: *Candida glabrata and C. albicans.*

The MI attributed to coinfection with bacteria and fungi in the overall studied population was 84.8% (Table 2) and was higher among male patients (53.8% vs. 46.2% for females) and those older than 70 years (66.7%). The MI due to bacterial infection was 94.9%, while it was 2.6% for fungal infections and 2.6% due to dual bacterial and fungal coinfections. In addition, the MI as a result of bacterial coinfection was 75.7% and 24.3% for multi- and monobacterial coinfections, respectively (*p* < 0.001). Based on sites of coinfection, the MI was 13.0% for upper respiratory infection, 24.1% for lower respiratory infection, and 63.0% for bacteremia. Moreover, levels of serum PCT, C-RP, ferritin, D-dimer, WBC count, and lymphocyte counts were higher in patients who died.

**Figure 2:**
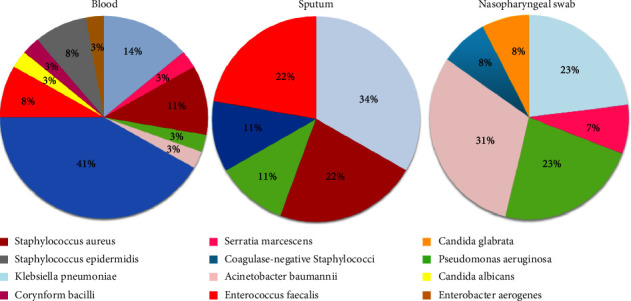
Bacterial and fungal coinfection distribution based on the type of specimen.

The patterns of bacterial and fungal coinfections based on the type of specimen where the microorganism was isolated are illustrated in Figure 2. The most numerous bacterial types were isolated from blood; coagulase-negative staphylococci had the highest proportion (41%), followed by *Klebsiella pneumoniae* (14%), *Staphylococcus aureus* (11%), and *Staphylococcus epidermidis* (8%). In sputum, the highest proportion of isolated bacteria was *Acinetobacter baumannii* (31%), followed by *Pseudomonas aeruginosa* and *Klebsiella pneumoniae* (23% for each). *Candida glabrata* was the sole cause of fungal coinfection in lower respiratory tract infections, with an 8% prevalence in the studied population. Regarding nasopharyngeal swabs, *Klebsiella pneumoniae* was considered the major isolated microorganism, with a 34% prevalence, followed by *Staphylococcus aureus* and *Enterococcus faecalis* (22% for each).

Table 3 shows the microorganism distribution among different specimen types based on sex. In females, the highest proportion of isolated microorganisms in sputum was *Klebsiella pneumoniae, Serratia marcescens, Acinetobacter baumannii,* and coagulase-negative staphylococci (25% for each), whereas coagulase-negative staphylococci had the highest prevalence of bacteria isolated from blood (36.8%). The highest proportions of isolated bacteria from nasopharyngeal swabs were *Klebsiella pneumoniae*, *Staphylococcus aureus*, and *Enterococcus faecalis*, with a 33.3% proportion for each. However, *Candida albicans* was the only fungus isolated from cerebrospinal fluids in both males and females. In males, the prevalence of *Pseudomonas aeruginosa* and *Acinetobacter baumannii* was higher in sputum (33.3% for each), followed by *Klebsiella pneumoniae* (22.2%). Similar to females, coagulase-negative staphylococci recorded in males had the highest prevalence of bacteria isolated from blood (47.1%). However, the higher proportion of isolated bacteria from nasopharyngeal swabs was *Klebsiella pneumoniae* (33.3%).

## 4. Discussion

Bacterial coinfection was common during previous respiratory viral pandemics. It was associated with a poor prognosis of the viral disease and considered a risk factor for death [23]. The MI of bacterial and fungal coinfection in COVID-19 patients was 50%, with more concern for antibiotic-resistant bacteria [3, 5]. The present data revealed that coinfections among COVID-19 in the ICU resulted from different types of bacteria and fungi, confirming what have been reported in other studies. Overall, coinfection among ICU COVID-19 patients results from bacteria [3], fungi [3], or other respiratory viruses [24]. In agreement with our findings, Silva et al. (2021) reported that the MI among COVID-19 patients in the ICU was 50.47% (83.14% being coinfected by fungi and/or bacteria) [25].

In addition, the present results found that the MI as a result of bacterial coinfection was 94.9%, 13.0% for upper respiratory infection, 24.1% for lower respiratory infection, and 63.0% for bacteremia. These results confirm what had been reported by Hughes et al., i.e., that bacteremia is considered a serious complication of bacterial superinfection and a risk factor for mortality in COVID-19 patients [10]. Bacteremia among COVID-19 patients has been reported worldwide with various percentages: 1.8% in Michigan City [26], 6% in New York City [27], 7.1% in the United Kingdom [10], 25% in Pennsylvania [28], and 37.0% in Milan [12], with mortality rates ranging between 9.8% and 47.5%. The worst-case scenario for critically ill COVID-19 patients is the development of polymicrobial coinfections [17], especially if one of the bacteria is multidrug-resistant, with a significant risk of complicating multiple organ failure and septic shock [8]. The main influence of bacterial coinfection in COVID-19 patients is mainly attributed to septicemia, multiorgan failure, septic shock, and respiratory, cardiac, kidney and liver dysfunction [27, 29, 30].

The present study shows an increase in inflammatory biomarkers, including PCT, C-RP, D-dimer, and ferritin, among patients who did not survive, in line with previous findings [27] and correlating significantly with respiratory failure distress, extended mechanical ventilation, and an increased MI rate [31]. Lymphocytopenia among our participants can be explained by interleukin upregulation and cytokine storms that cause lymphocyte apoptosis [7, 9], which have been considered risk factors for coinfection during the COVID‐19 pandemic [6].

Antibiotic resistance was detected among approximately half of the patients who died (51.3%), which creates additional challenges in controlling patient outcomes and prognosis [14]. The prophylactic use of broad-spectrum antibiotics among such patients must be taken into consideration to avoid the drawbacks of long-term broad-spectrum antibiotic misuse [28]. The protocols regarding broad-spectrum antibiotic use are different at the national level; for example, the Chinese Institutes of Health do not recommend the use of broad-spectrum antibiotics even in critically ill patients without bacterial coinfection [17], whereas in the Netherlands, empirical antibiotics are recommended for all ICU and mechanically ventilated patients [32].

Among the study strengths, this study is the first in Jordan and included accurately identified bacterial and fungal isolates that caused coinfection among COVID-19 patients in the ICU, and it is the first study to clinically characterize ICU COVID-19 patients in the Middle East and North Africa region (MENA). Additionally, the samples were obtained from different types of specimens. Among limitations, this was a single-center study, and the present findings should be considered with caution. The small sample size is attributed to the nature of the studied population, namely, ICU patients.

In conclusion, bacterial/fungal coinfections hurt critically ill COVID-19 patients and may lead to worse disease prognosis and increased MI. Timely prediction of bacterial and fungal coinfection among critically ill ICU patients through monitoring of inflammatory biomarkers before microbiological culture and antibiotic susceptibility results may play a critical role in improving patient prognosis and increasing the survival rate.

## Figures and Tables

**Table 1:** General characteristics of bacterial and/or fungal coinfections among ICU COVID-19 patients.

Patient #	Isolated microorganisms (type of specimen)	Gender	Age	Patient outcomes
1	*Klebsiella pneumoniae* (sputum and nasopharyngeal swab) *Serratia marcescens* (blood)	Male	72	Died
2	*Klebsiella pneumoniae* (blood)	Female	66	Died
3	*Staphylococcus aureus* (nasopharyngeal swab) *Pseudomonas aeruginosa* (nasopharyngeal swab)	Male	56	Died
4	*Acinetobacter baumannii* (sputum) Coagulase-negative staphylococci (sputum) *Serratia marcescens* (sputum)	Female	63	Died
5	Coagulase-negative staphylococci (blood)	Female	85	Died
6	Coagulase-negative staphylococci (blood)	Female	85	Died
7	*Enterococcus faecalis* (pus)	Male	66	Died
8	*Klebsiella pneumoniae* (blood)	Female	28	Died
9	Coagulase-negative staphylococci (blood) *Staphylococcus aureus* (blood) *Enterococcus faecalis* (blood) *Klebsiella pneumoniae* (blood)	Male	79	Died
10	Coagulase-negative staphylococci (blood) *Pseudomonas aeruginosa* (blood)	Male	89	Died
11	*Candida glabrata* (sputum)	Male	61	Died
12	*Pseudomonas aeruginosa* (sputum)	Male	79	Survived
13	*Pseudomonas aeruginosa* (sputum)	Male	77	Died
14	*Staphylococcus aureus* (blood)	Female	29	Died
15	Coagulase-negative staphylococci (blood, and nasopharyngeal swab)	Male	83	Died
16	*Klebsiella pneumoniae* (nasopharyngeal swab)	Female	76	Survived
17	Coagulase-negative staphylococci (blood)	Female	82	Died
18	*Staphylococcus aureus* (blood) *Klebsiella pneumoniae* (blood)	Female	85	Died
19	Coagulase-negative staphylococci (blood)	Male	77	Died
20	Coagulase-negative staphylococci (blood)	Male	78	Survived
21	*Klebsiella pneumoniae* (sputum)	Male	55	Died
22	*Pseudomonas aeruginosa* (sputum)	Male	78	Died
23	*Acinetobacter baumannii* (blood) *Klebsiella pneumoniae* (blood)	Female	15	Died
24	Coagulase-negative staphylococci (blood)	Female	80	Died
25	*Klebsiella pneumoniae* (blood)	Male	77	Died
26	*Candida albicans* (cerebrospinal fluid)	Female	82	Survived
27	*Enterococcus faecium* (blood)	Female	64	Died
28	*Coryneform bacilli* (blood)	Female	67	Died
29	*Staphylococcus epidermidis* (blood)	Male	57	Died
30	*Candida albicans* (blood) Coagulase-negative staphylococci (blood)	Female	80	Died
31	*Acinetobacter baumannii* (sputum)	Male	74	Died
32	Coagulase-negative staphylococci (blood)	Female	84	Died
33	*Acinetobacter baumannii* (sputum) *Klebsiella pneumoniae* (sputum)	Male	83	Died
34	*Staphylococcus epidermidis* (blood)	Female	80	Died
35	Coagulase-negative staphylococci (blood)	Male	83	Died
36	*Enterococcus faecalis* (nasopharyngeal swab)	Female	29	Died
37	*Enterococcus faecalis* (nasopharyngeal swab)	Male	89	Died
38	*Staphylococcus aureus* (nasopharyngeal swab)	Female	86	Died
39	*Staphylococcus epidermidis* (blood)	Male	87	Died
40	Coagulase-negative staphylococci (blood)	Female	29	Survived
41	*Klebsiella pneumoniae* (nasopharyngeal swab)	Male	78	Survived
42	*Enterococcus faecium* (blood)	Female	66	Survived
43	Coagulase-negative staphylococci (blood)	Male	76	Died
44	*Enterobacter aerogenes* (blood) Coagulase-negative staphylococci (blood)	Male	89	Died
45	*Staphylococcus aureus* (blood)	Female	88	Died
46	*Acinetobacter baumannii* (sputum)	Male	82	Died

**Table 2:** Impact of bacterial and/or fungal coinfections among ICU COVID-19 patients.

Variable				Patient's outcomes		*p* value*∗*
				Survived	Died	
Sex	Male			42.9%	53.8%	0.466
	Female			57.1%	46.2%	

Age	Less than 29 years			0.0%	5.1%	0.642
	29–49 years			14.3%	5.1%	
	50–65 years			0.0%	15.4%	
	66–70 years			14.3%	7.7%	
	More than 70 years			71.4%	66.7%	

Cause of coinfections	Fungi (sole)			14.3%	2.6%	0.366
	Dual (bacterial and fungal coinfections)			0.0%	2.6%	
	Bacteria			85.7%	94.9%	
	Monobacterial			0.0%	24.3%	<0.001
	Multibacterial			100.0%	75.7%	

Antimicrobial susceptibility test	Antibiotic resistance		Yes	28.6%	51.3%	0.149
			No	71.4%	48.7%	
	Resistance to antibiotics	Oxacillin resistance	Yes	28.6%	38.5%	0.618
			No	71.4%	61.5%	
		Methicillin resistance	Yes	14.3%	15.4	0.268
			No	85.7%	84.6%	

Inflammatory biomarkers (mean ± SD)	Procalcitonin (g/dl)			0.93 ± 0.47	22.77 ± 0.87	0.585
	C-reactive protein (mg/L)			48.15 ± 0.36	95.88 ± 0.93	0.404
	Ferritin (ng/ml)			838.18 ± 0.98	4230.96 ± 0.78	0.692
	D-dimer (ng/ml)			607.00 ± 2.03	9200.40 ± 7.50	0.446
	White blood cells count (×10^9^ cells/L)			12.63 ± 5.12	18.73 ± 3.48	0.586
	Neutrophils counts (%)			89.75 ± 1.26	81.48 ± 7.89	0.326
	Lymphocyte counts (%)			5.50 ± 1.53	13.07 ± 2.54	0.356

Site of coinfection	Upper respiratory infection			28.6%	13.0%	0.171
	Lower respiratory infection			14.3%	24.1%	
	Bacteremia			42.9%	63.0%	
	Meningitis			14.3%	0.0%	

Total				15.2%	84.8%	—

Normal range: procalcitonin: less than 0.5 ng/ml: low risk of severe sepsis, 0.5–2.0 ng/ml: clinical suspicion of sepsis, more than 2.0–10 ng/ml: high risk of severe sepsis, more than 10 ng/ml: high likelihood of septic shock; C-reactive protein: less than 5.0 mg/L; ferritin: male: 22–322 ng/ml, female: 10–291 ng/ml; D-dimer: less than 500 ng/ml; globulin: 2–3 g/dl; white blood cell count: 4.5–11 × 10^9^/L; neutrophil count: 30–75%; lymphocytes: 20–40%. ^*∗*^Statistically significant at *p* < 0.05.

**Table 3:** Microorganism distribution among different specimen types based on sex.

Isolated microorganism	Type specimen				Total (%)
	Sputum	Blood	Nasopharyngeal swab	Cerebrospinal fluid	
^$^ * Females*					
*Klebsiella pneumoniae*	25.0%	15.8%	33.3%	0.0%	18.5
*Serratia marcescens*	25.0%	0.0%	0.0%	0.0%	3.7
*Staphylococcus aureus*	0.0%	15.8%	33.3%	0.0%	14.8
*Acinetobacter baumannii*	25.0%	5.3%	0.0%	0.0%	7.4
Coagulase-negative staphylococci	25.0%	36.8%	0.0%	0.0%	29.6
*Enterococcus faecalis*	0.0%	10.5%	33.3%	0.0%	11.1
*Candida albicans*	0.0%	5.3%	0.0%	100.0%	7.4
*Coryneform bacilli*	0.0%	5.3%	0.0%	0.0%	3.7
*Staphylococcus epidermidis*	0.0%	5.3%	0.0%	0.0%	3.7

^*∗*^ * Males*					
*Klebsiella pneumoniae*	22.2%	11.8%	33.3%	0.0%	18.2
*Serratia marcescens*	0.0%	5.9%	0.0%	0.0%	2.9
*Staphylococcus aureus*	0.0%	5.9%	16.7%	0.0%	5.9
*Pseudomonas aeruginosa*	33.3%	5.9%	16.7%	0.0%	14.7
*Acinetobacter baumannii*	33.3%	0.0%	0.0%	0.0%	8.8
Coagulase-negative staphylococci	0.0%	47.1%	16.7%	0.0%	26.5
*Enterococcus faecalis*	0.0%	5.9%	16.7%	100.0%	8.8
*Candida glabrata*	11.1%	0.0%	0.0%	0.0%	2.9
*Staphylococcus epidermidis*	0.0%	11.8%	0.0%	0.0%	5.9
*Enterobacter aerogenes*	0.0%	5.9%	0.0%	0.0%	2.9

^$^ *p* value was 0.329; ^*∗*^*p* value was 0.246.

## Data Availability

The database analyzed for the current study is available from the corresponding author upon reasonable request.
